# Association between Extract Rheum rhaponticum 731 (ERr 731) prescription and subsequent breast cancer

**DOI:** 10.1007/s10549-025-07711-9

**Published:** 2025-05-13

**Authors:** Peter W. Heger, Dirk Hotz, Matthias Kalder, Karel Kostev

**Affiliations:** 1 Health Research Services GmbH, Hofaeckerstr. 14, 76698 Ubstadt-Weiher, Germany; 2 Department of Gynecology and Obstetrics, Philipps-University, Marburg, Germany

**Keywords:** ERr 731, Rhapontic rhubarb, Phytotherapy, Breast cancer, Association, Germany

## Abstract

**Aims:**

The special extract ERr 731 from the roots of rhapontic rhubarb has been prescribed for women with menopausal symptoms for more than 30 years. The aim of the present study is to evaluate the association between ERr 731 therapy and subsequent breast cancer in women in a real-world setting. ERr 731 users were compared to women without this therapy as well as women receiving hormone therapy.

**Methods:**

This retrospective cohort study included data of women treated by 260 office-based gynecologists in Germany who received a prescription for ERr 731 between 1993 and 2022 (IQVIA Disease Analyzer database). These women were matched to women without ERr 731 prescriptions as well as women with hormone replacement therapy (HRT) prescriptions (1:3) using nearest neighbor propensity scores. A univariate Cox regression analysis was conducted to evaluate the associations between ERr 731 prescription and breast cancer risk compared to women without ERr 731 prescription and women with HRT prescriptions.

**Results:**

A total of 5,686 women with versus 17,058 women without ERr 731 prescription were available for the first analysis, and 2,616 women with ERr 731 prescription (a proportion of the 5,686 women used in the first analysis) and 7,848 women with HRT prescriptions for the second (average age 52–53 years). ERr 731 was not associated with an increased risk of breast cancer diagnosis when the group of women with ERr 731 prescription was compared to women without (OR: 1.01, 95% CI: 0.81–1.26) or to that of women with HRT prescription ((OR: 0.96, 95% CI: 0.69–1.33). No associations were observed in age-stratified analyses or in women with and without menopausal or other perimenopausal disorders.

**Conclusion:**

The present study provides strong evidence that ERr 731 is not associated with an increased risk of breast cancer diagnosis compared to both non-users and HRT users. Given its favorable safety profile, ERr 731 may represent a viable alternative to HRT, particularly for women concerned about breast cancer risk.

## Introduction

Menopausal and perimenopausal disorders affect millions of women worldwide, often causing a range of symptoms such as hot flashes, mood swings, sleep disturbances, and bone density loss [1, 2]. Hormone replacement therapy (HRT) is a common treatment for these symptoms but carries potential risks, including an increased risk of breast cancer and cardiovascular events [3]. As a result, many women seek alternative, non-hormonal therapies to manage their symptoms effectively and safely [4].

Rheum rhaponticum, commonly known as rhapontic rhubarb, is a plant with a long history of ethnomedicinal use. A number of published scientific studies have explored its phytochemical composition, biological activities, and therapeutic potential, particularly in relation to menopausal symptoms and the plant’s anti-inflammatory, antioxidant, and antimicrobial properties [5–10]. In Germany, the special extract ERr 731 from the roots of rhapontic rhubarb has been prescribed regularly for women with menopausal symptoms since 1993. The extract has been marketed as Phytoestrol N, Phyto-Strol, Phyto-Strol Loges, and Phyto-Strol compact and as femiLoges [11].

The updated S3 guideline on peri- and postmenopause includes the use of phytoestrogen-containing plants including ERr 731 for menopausal symptoms [12, 13]. During the consensus process for the preparation of the new edition of the guideline, it became apparent that a sufficient evidence base had been established for ERr 731. The supporting research included one observational study [14], two randomized clinical trials [6, 15], two long term (one and two years) [16], and one post-marketing study which registered approx. 200 adverse events with approx. 155 million daily doses administered [11]. In an open-label prospective study including 129 perimenopausal women in India conducted one year later, Shah et al. reported a significant reduction in the mean menopause rating scale (MRS) II score after 12 weeks of therapy with ERr 731. Researchers have also observed significant reductions in mean blood glucose and glycated hemoglobin level at 12 weeks [17]. In terms of safety, ERr 731 therapy has previously been shown not to cause serious adverse events. For example, in the work of Chang et al. [11] complaints were rarely reported and were limited to gastrointestinal symptoms, which were well tolerated.

Furthermore, no clinically relevant changes in different parameters, including endometrial biopsies or bleeding, have been observed in clinical trials for ERr 731 [6, 14, 16, 18]. Hasper et al. suggest that the treatment is safe for breast and endometrial tissue because it does not increase progesterone or 17β-estradiol levels [16].

Nevertheless, to date, no studies involving large numbers of patients from real-world care settings have explored the safety of ERr 731 in terms of breast cancer risk. The aim of the present study is to evaluate the association between ERr 731 therapy and subsequent breast cancer in women in a real-world setting.

## Methods

### Data source

This analysis used electronic medical records from the IQVIA™ Disease Analyzer database, which contains case-based information provided by nearly 3,000 office-based physicians (both GPs and specialists) in Germany. The sample of practices included is geographically representative for Germany, covering eight major German regions. Analyses conducted using data from the database did not indicate any lack of representativeness or validity in comparison with reference statistics. The database appears to be suitable for pharmaco-epidemiological and pharmaco-economic studies [19]. IQVIA ensures the accuracy, consistency, and completeness of the data. Evident data limitations are communicated immediately by IQVIA, ensuring that analyses can be adapted accordingly.

## Ethics approval

German law permits the use of anonymized, de-identified electronic medical records for research purposes. In Germany, it is not necessary to obtain informed consent from patients or approval from a medical ethics committee for this type of observational study that contains no directly identifiable data. Therefore, no waiver of ethical approval can be obtained from an Institutional Review Board (IRB) or ethics committee. The company and the authors involved had no access to any identifying information at any moment during the data analysis.

### Study design and population

The current study is a retrospective cohort study that included data from two cohorts. The first cohort included women treated by one of 260 office-based gynecologists in Germany who received a first prescription for ERr 731 between 1993 and 2022 (index date). Women were only included if they had at least 12 months of observation time prior to the index date and at least six months of follow-up time after the index date. Patients with a documented cancer diagnosis prior to, on or within six months after the index date were excluded. A minimum period of six months starting from the index date was considered for each patient, as this time was needed to allow the prescription of ERr 731 to take effect.

The second cohort contained data from women without prescriptions for ERr 731. A matched-pair design was used to avoid selection bias and mitigate the impact of confounding variables on the outcomes. Women with ERr 731 prescriptions were matched to women without these prescriptions in a 1:3 ratio using nearest neighbor propensity scores. Matching was based on age, health insurance status (private versus statutory), and co-diagnoses documented within 12 months prior to or on the index date, including obesity (ICD-10: E66), menopausal, and other perimenopausal disorders (ICD-10: N95), and diseases of the breast (ICD-10: N60–N64), as well as prescriptions for hormone replacement therapy (HRT) throughout the entire study period. A standardized mean difference (SMD) of less than 0.1, indicating that adequate covariate balance had been achieved, was considered acceptable [20].

As a sensitivity analysis, one further cohort was selected. This comprised women receiving hormone replacement therapy (HRT)—estrogen or progesterone—between 1993 and 2022. As in the second cohort, these women had to have at least 12 months of observation time prior to the index date and at least six months of follow-up time after the index date. They also had to have no cancer diagnosis prior to or on the index date in order to be included. Finally, women undergoing HRT therapy were matched to women with ERr 731 prescriptions (1:3) using nearest neighbor propensity scores based on age, health insurance status, and co-diagnoses.

Participant data used in the sensitivity analysis (both with ERr 731 prescription and HRT prescription) constituted a small portion of the main data of participants included in the previous analysis.

### Statistical analyses

The differences between baseline characteristics of women with and without ERr 731 prescription were compared using T-test for age and Chi squared test for categorical variables. A univariate Cox regression analysis was conducted to evaluate the associations between ERr 731 prescriptions and breast cancer diagnoses as compared to women without ERr prescriptions and women with HRT prescriptions from seven months to up to ten years after the index date. The results of the Cox regression analysis are displayed as Hazard Ratios (HR) with 95% confidence intervals. Regression analyses were also conducted separately by age group. A *p*-value of < 0.05 was considered statistically significant. All analyses were performed using SAS Version 9.4 (SAS Institute, Cary, USA).

## Results

### Comparison of ERr 731 and non-ERr 731 cohorts

**Fig. 1:**
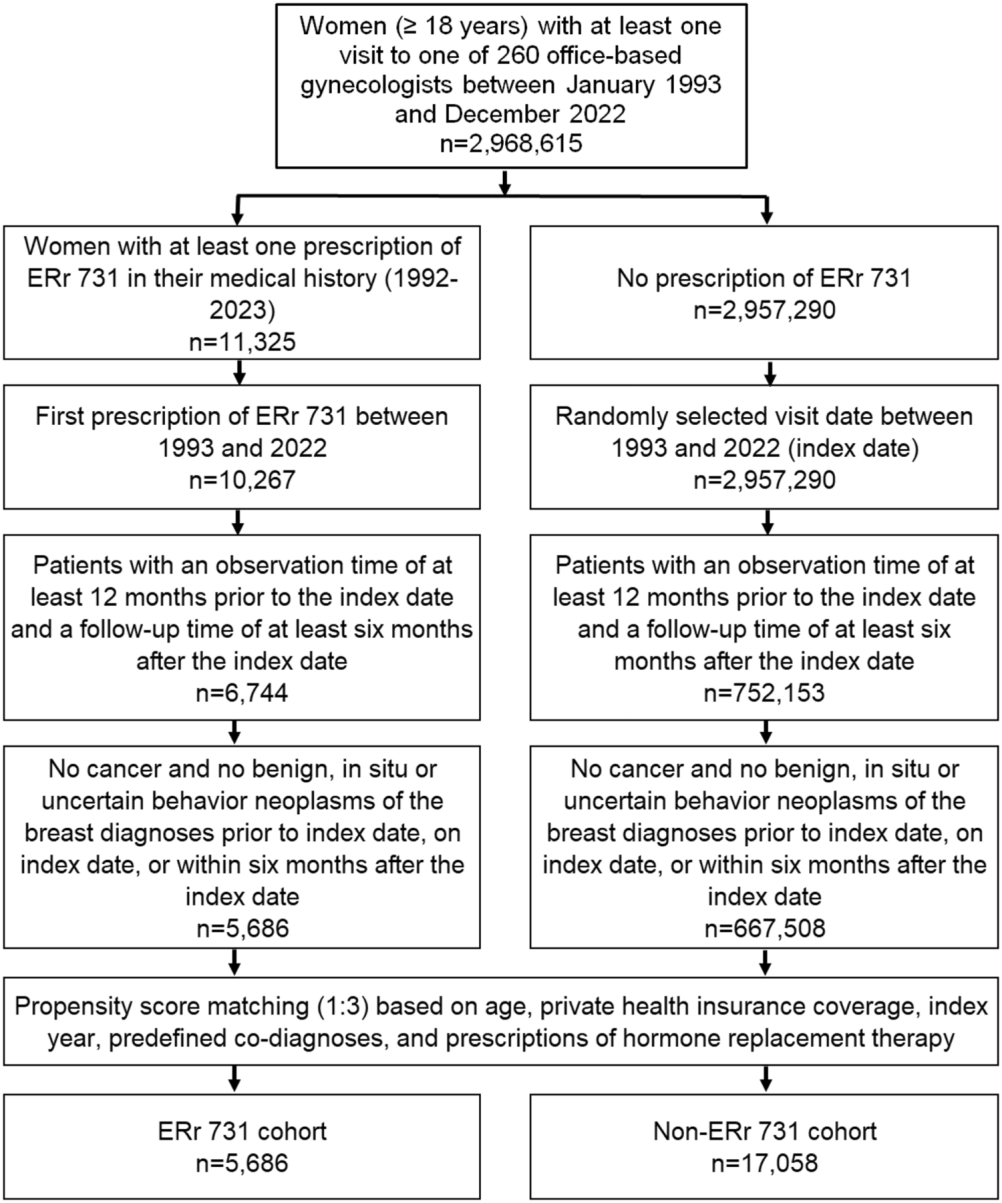
Selection of study patients (patients with ERr 731 prescription versus patients without ERr 731 prescription)

Out of 2,968,615 adult women with at least one visit to one of 260 office-based gynecologists, ERr 731 was prescribed to 10,267 women between 1992 and 2022. After excluding patients who lacked the required minimum 12 months of pre-observation time before the index date and at least six months of follow-up time after the index date, 6,744 women with ERr 731 prescriptions remained. When women with diagnoses of cancer or benign, in situ, or uncertain behavior neoplasms of the breast prior to, on, or within six months after the index date were excluded, some 5,686 women with ERr 731 prescriptions were available for analysis. For each of the 5,686 women treated with ERr 731, we identified three matched controls, resulting in 17,058 matched patients without ERr 731 prescriptions for analysis (Fig. 1).

Table 1 presents the baseline characteristics of the study patients. Due to the matched-pair process, there were no significant differences between the ERr 731 and non-ERr 731 cohorts in terms of age (53 years), obesity (9%), menopausal and other perimenopausal disorders (81%), breast diseases (43%), and HRT prescriptions (50%). The proportion of privately insured patients was slightly but significantly higher among ERr 731 users (11.8% vs. 8.7%) (Table 1).

**Table 1:** Basic characteristics of study patients (Patients with ERr 731 prescription versus patients without ERr 731 prescription)

Variable	Patients with ERr 731 prescription	Patients without ERr 731 prescription	SMD	*P*-value
N	5,686	17,058		
Age (mean, SD)	52.6 (6.6)	52.5 (6.8)	0.015	0.504
18–40 years (*N*, %)	111 (2.0)	412 (2.4)		0.240
41–50 years (*N*,%)	2,003 (35.2)	6,005 (35.2)		
51–60 years *N*, (%)	3,015 (53.0)	8.959 (52.5)		
> 60 years (*N*, %)	557 (9.8)	1,682 (9.8)		
Private health insurance coverage (*N*, %)	671 (11.8)	1,482 (8.7)	−0.031	0.001
Obesity (*N*, %)	501 (8.8)	1,556 (9.1)	0.003	0.479
Menopausal and other perimenopausal disorders (*N*, %)	4,596 (80.8)	13,795 (80.9)	< 0.001	0.946
Diseases of breast (*N*, %)	2,467 (43.4)	7,338 (43.0)	−0.004	0.626
Index year 1993–2010 (*N*, %)	620 (10.9)	1,860 (10.9)	0.004	0.971
Index year 2011–2015 (*N*, %)	1,544 (27.2)	4,643 (27.2)		
Index year 2016–2019 (*N*, %)	1,971 (34.7)	5,956 (34.9)		
Index year 2019–2022 (*N*, %)	1,551 (27.3)	4,599 (27.0)		
Prescriptions of HRT (*N*, %)	2,855 (50.2)	8,588 (50.4)	0.001	0.860

**Fig. 2:**
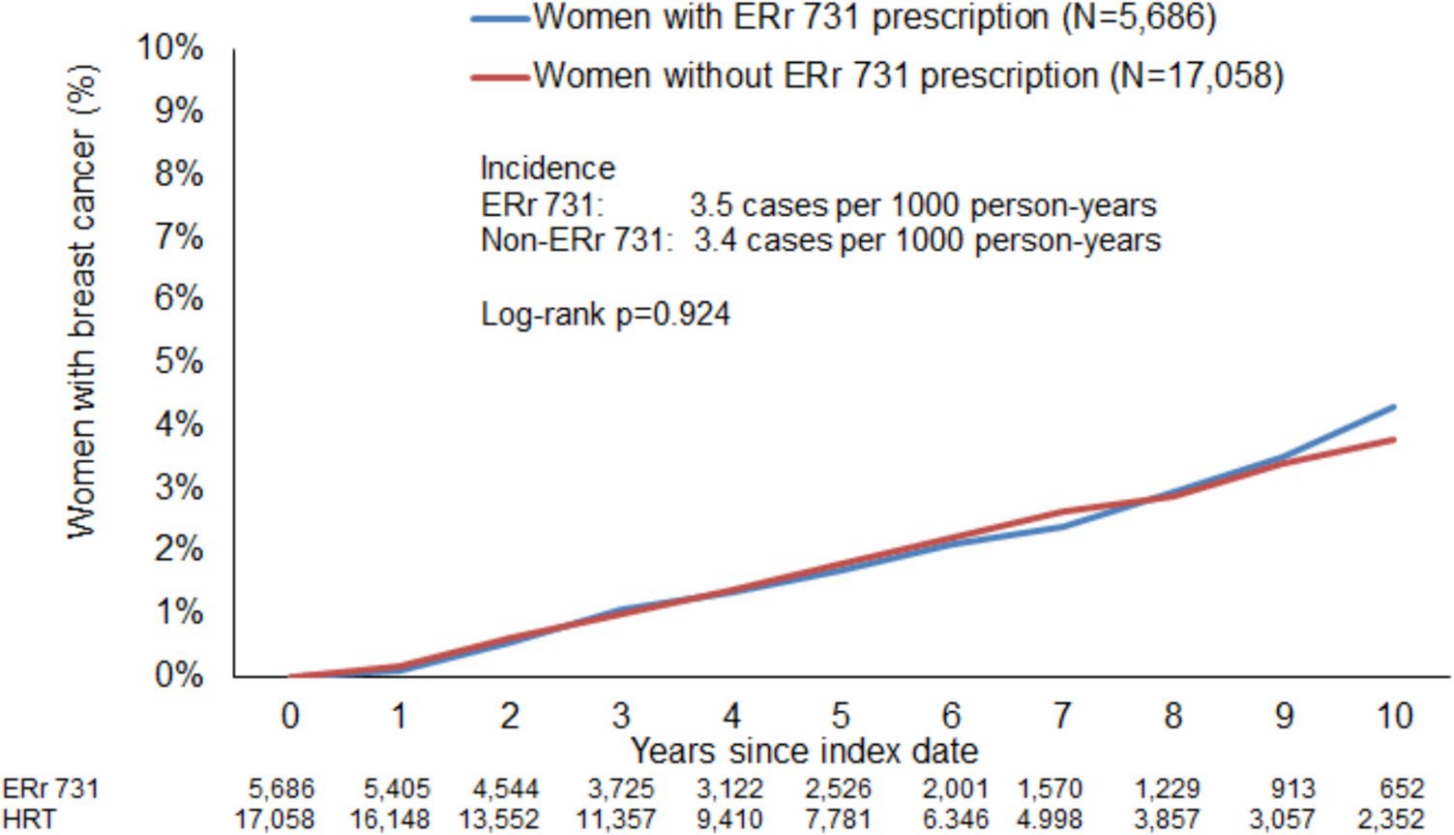
Incidence of new breast cancer diagnoses stratified by study cohort (Patients with ERr 731 prescription versus patients without ERr 731 prescription)

Figure 2 shows the Kaplan–Meier curves for incident breast cancer diagnosis. The incidence was 3.5 cases per 1,000 person-years in the ERr 731 cohort and 3.4 cases per 1,000 person-years in the non-ERr 731 cohort (*p*-value = 0.924).

Regression analysis confirmed that there was no significant difference between the study cohorts. ERr 731 prescription was not associated with an increased risk of breast cancer diagnosis (OR: 1.01, 95% CI: 0.81–1.26) (Table 2). No association was observed in the age-stratified analyses or in women with and without menopausal or other perimenopausal disorders (Table 2).

**Table 2:** Association between ERr 731 prescription and breast cancer diagnosis from 6 months after up to 10 years after the index date (Cox regression)

Patient group	Incidence (cases per 1,000 person-years) in ERr 731 cohort	Incidence (cases per 1,000 person-years) in non-ERr 731 cohort	Hazard Ratio (95% CI)	*P*-value
Total	3.5	3.4	1.01 (0.81–1.26)	0.924
18–40 years	0.0	1.6	–	
41–50 years	2.5	2.7	0.90 (0.58–1.40)	0.649
51–60 years	3.9	3.7	1.05 (0.79–1.41)	0.731
> 60 years	5.5	4.8	1.12 (0.64–1.96)	0.684
Women with menopausal and other perimenopausal disorders	3.6	3.5	1.03 (0.81–1.32)	0.794
Women without menopausal and other perimenopausal disorders	2.8	3.0	0.90 (0.51–1.60)	0.723

### Comparison of ERr 731 with hormone replacement therapy

**Fig. 3:**
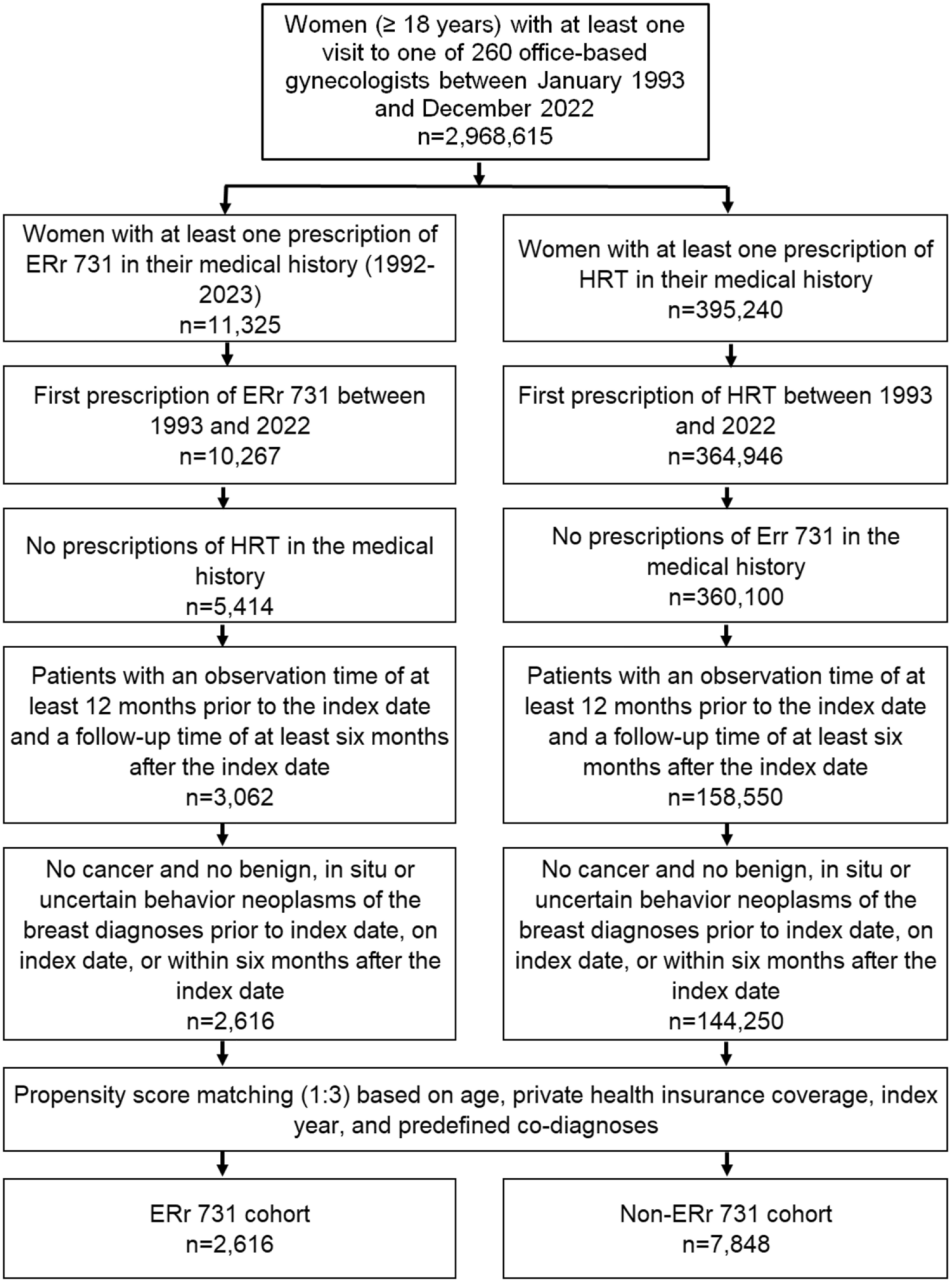
Selection of study patients (Patients with ERr 731 prescription versus patients with HRT prescription)

Between 1992 and 2022, ERr 731 was prescribed to 10,267 women and HRT to 384,946 women for the first time. After excluding women with HRT prescription from the ERr 731 cohort and those with ERr 731 prescription from the HRT cohort, 5,414 women with ERr 731 and 360,100 women with HRT prescription remained. When we excluded patients lacking at least 12 months of pre-observation time prior to the index date and at least six months of follow-up time after the index date, 3,082 women with ERr 731 and 158,550 women with HRT prescription were available for analysis. After further excluding women with diagnoses of cancer or benign, in situ, or uncertain behavior neoplasms of the breast prior to, on, or within six months after the index date, a total of 2,616 women with ERr 731 prescription and 144,250 with HRT prescription remained. We identified three matched HRT patients for each of the 2,616 women treated with ERr 731, resulting in 7,848 matched patients with HRT prescriptions being available for analysis (Fig. 3).

Table 3 presents the baseline characteristics of the study patients. Due to the matched-pair process, there were no significant differences between the ERr 731 and HRT cohorts in terms of age (52 years), private insurance status (10–11%), obesity (10%), menopausal and other perimenopausal disorders (76%), or breast diseases (38%) (Table 3).

**Table 3:** Basic characteristics of study patients (Patients with ERr 731 prescription versus patients with HRT prescription)

Variable	Patients with ERr 731 prescription	Patients with HRT prescription	SMD	*P*-value
N	2,616	7,848		
Age (mean, SD)	52.1 (6.3)	52.1 (6.3)	0.002	0.962
18–40 years (*N*, %)	60 (2.3)	179 (2.3)		0.999
41–50 years (*N*,%)	948 (36.2)	2,848 (36.3)		
51–60 years *N*, (%)	1,415 (54.1)	4,248 (54.1)		
> 60 years (*N*, %)	193 (7.4)	573 (7.3)		
Private health insurance coverage (*N*, %)	285 (10.9)	791 (10.1)	−0.008	0.243
Obesity (*N*, %)	269 (10.3)	786 (10.0)	−0.003	0.694
Menopausal and other perimenopausal disorders (*N*, %)	1,979 (75.7)	5,938 (75.7)	0.001	0.990
Diseases of breast (*N*, %)	987 (37.7)	2,942 (37.5)	−0.002	0.823
Index year 1993–2010 (*N*, %)	256 (9.8)	767 (9.8)	−0.002	0.999
Index year 2011–2015 (*N*, %)	688 (26.3)	2,062 (26.3)		
Index year 2016–2019 (*N*, %)	924 (35.3)	2,767 (35.2)		
Index year 2019–2022 (*N*, %)	748 (28.6)	2,252 (28.7)		

**Fig. 4:**
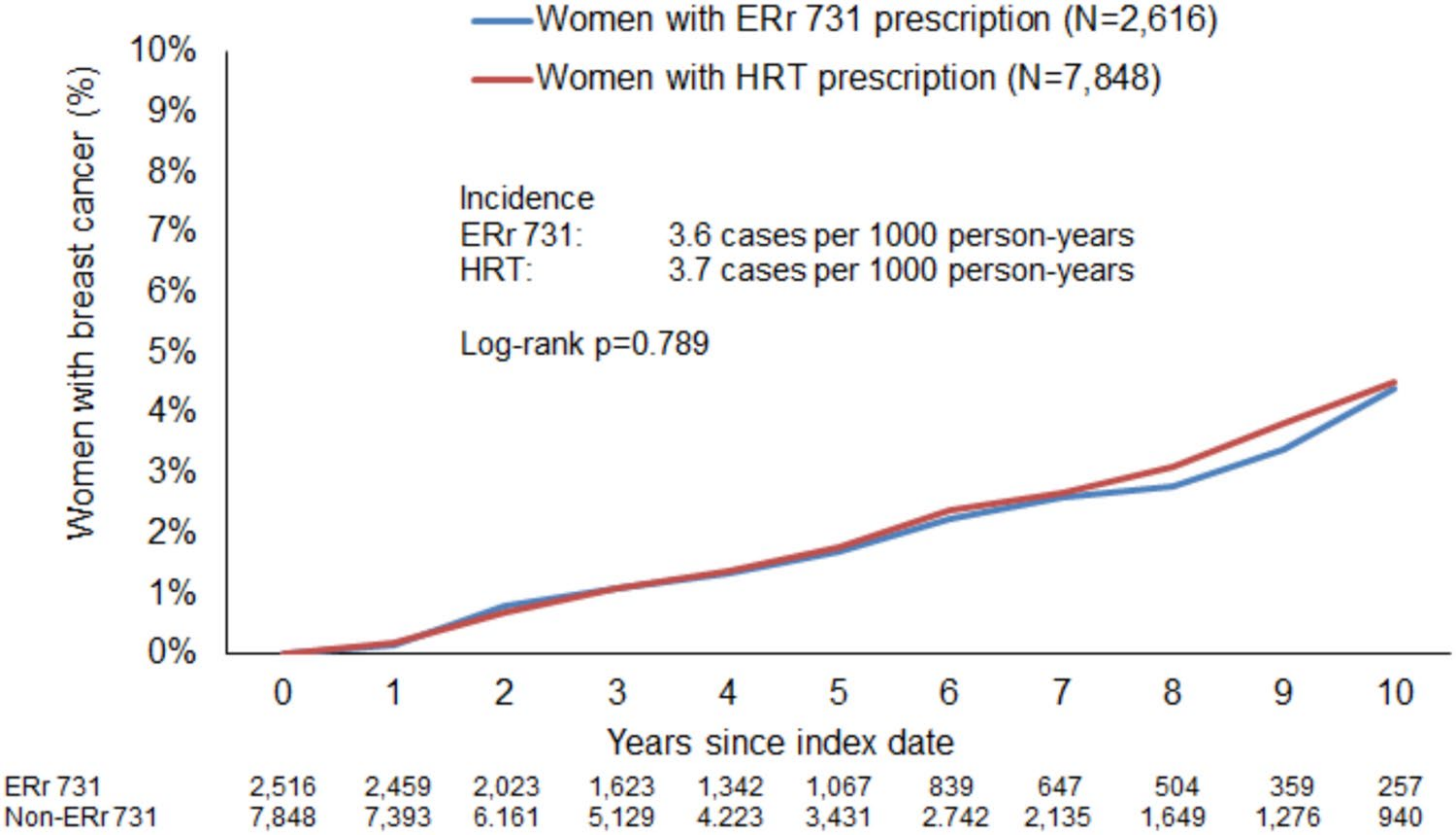
Incidence of new breast cancer diagnoses stratified by study cohort (patients with ERr 731 prescription versus patients with HRT prescription)

Figure 4 shows the Kaplan–Meier curves for incident breast cancer diagnosis. The incidence was 3.6 cases per 1,000 person-years in the ERr 732 cohort and 3.7 cases per 1,000 person-years in the HRT cohort (*p*-value 0.789).

Regression analysis confirmed that there was no significant difference between the study cohorts. ERr 731 prescriptions were not associated with an increased risk of breast cancer diagnosis (HR: 0.96, 95% CI: 0.69–1.33) (Table 2). No association was observed in the age-stratified analyses or among women with and without menopausal or other perimenopausal disorders (Table 4).

**Table 4:** Association between ERr 731 prescription and breast cancer diagnosis from 6 months after up to 10 years after the index date (Cox regression) (Patients with ERr 731 prescriptions versus patients with HRT prescriptions)

Patient group	Incidence (cases per 1,000 person-years) in ERr 731 cohort	Incidence (cases per 1,000 person-years) in HRT cohort	Hazard ratio (95% CI)	*P*-value
Total	3.6	3.7	0.96 (0.69–1.33)	0.791
18–40 years	0.0	3.5	–	
41–50 years	2.4	3.0	0.79 (0.41–1.52)	0.477
51–60 years	4.5	4.1	1.09 (0.73–1.64)	0.676
> 60 years	4.0	4.5	0.88 (0.29–2.67)	0.817
Women with menopausal and other perimenopausal disorders	3.9	3.9	0.99 (0.69–1.42)	0.945
Women without menopausal and other perimenopausal disorders	2.7	3.1	0.83 (0.38–1.81)	0.637

## Discussion

To the best of the authors’ knowledge, this is the first real-world study to examine the association between ERr 731 and breast cancer risk in women of different age groups.

The study results as presented focus on the potential relationship between the prescription of ERr 731 and the incidence of breast cancer in different cohorts. The study findings suggest that ERr 731 is not associated with a higher risk of breast cancer compared to women not receiving the treatment or women using HRT.

These findings are not surprising. Both estrogen receptor-(ER)independent and ER-dependent effects may influence the development of breast cancer [21].

Investigations focusing on ERr 731 and its compounds have shown that they activate and bind to ERβ in different cell lines with high specificity [22, 23]. ERr 731 is primarily composed of rhaponticin (which makes up nearly 90% of the treatment, along with smaller amounts (about 5% of its aglycones and desoxyrhaponticin. In plants, these natural hydroxystilbene compounds are produced through the same biosynthetic pathway as resveratrol. ERr 731 acts in a manner similar to estrogens, but has been identified as a highly selective agonist of the estrogen receptor (ER β [9]). The extract ERr731 does not mimic estrogen-stimulated growth of MNU-induced breast cancer in ovariectomized Sprague–Dawley rats [24]. Rheum rhaponticum (ERr 731 has been shown not to induce cell proliferation of MCF-7 or MDA-MB-231 cells [25]. Its clinical safety has been demonstrated in a number of human studies [11, 26]. Furthermore, a 2009 German case–control study showed for the first time that herbal preparations (including Rheum rhaponticum) used to treat climacteric disorders may potentially protect against invasive breast cancer [27]. Some authors have previously discussed breast cancer-related effects driven by ER-α activation [28, 29]. ERr 731 treatment is not associated with such risks, probably because it lacks ER-α agonistic activity [9, 22].

The results of the present study may have important clinical implications for the management of menopausal symptoms. As a non-hormonal, plant-based therapy, ERr 731 has been gaining popularity due to its purported efficacy in alleviating vasomotor symptoms such as hot flashes and night sweats, which are commonly experienced by menopausal women [7]. Given the widespread use of HRT and the associated concerns about its long-term safety, particularly in terms of breast cancer risk [30, 31] it is crucial to evaluate the safety of alternative therapies such as ERr 731.

This finding is particularly relevant for women at elevated risk of breast cancer, who are typically advised against HRT due to its estrogenic activity. While the results suggest that ERr 731 may offer symptom relief without a corresponding rise in breast cancer risk, these conclusions should be interpreted within the context of observational data and potential residual confounding. Notably, the lack of significant variation in breast cancer risk across age brackets and clinical subgroups—such as women with menopausal or perimenopausal disorders—supports the potential utility of ERr 731 across diverse patient populations. These results may inform clinical decision making, particularly in cases where non-hormonal treatment options are prioritized. However, further controlled studies are warranted to confirm these associations and gain a better understanding of long-term safety profiles.

Despite the strengths of the study, including the large real-world samples available for analysis, use of matched cohorts, and the robust statistical analyses, there are also some limitations that warrant consideration. First, only patients who had received ERr 731 prescriptions from gynecologists were selected for analyses, as ERr 731 was a prescription-only drug until 2005. After 2005, however, it became an OTC drug like other herbal medicines in Germany, and therefore, did not require a prescription. As the database does not include data on the use of herbal medicines purchased without prescription by patients, a proportion of women taking ERr 731 may unintentionally have been excluded from the present study. Consequently, we do not have valid information on the total therapy duration for ERr 731. The second limitation is the potential for misclassification of breast cancer diagnoses. Although the study used validated data sources for diagnosis information, it is possible that some cases were missed or classified incorrectly. All diagnosis assessments rely on ICD codes entered by gynecologists, which do not distinguish between severities or outcomes. Third, the database used does not contain information on socioeconomic status or lifestyle-related risk factors (smoking, alcohol, and physical activity) and biochemical markers for breast cancer or on conditions resulting in hormonal fluctuations. These conditions have a significant influence on the risk of breast cancer progression, which may be an important confounder in the investigation of breast cancer risk. Fourth, the database includes patients observed in office-based practices and cannot link patients with hospitals.

The matching process mitigates potential biases that could otherwise have influenced the study outcomes. For instance, obesity is a known risk factor for breast cancer, especially in postmenopausal women, as it increases estrogen levels through the peripheral conversion of androgens in adipose tissue [32]. By ensuring comparable obesity rates between the groups (9–10%), the study reduces the likelihood that differences in breast cancer incidence would be confounded by this factor. In addition, the similar rates of menopausal and perimenopausal disorders across groups (81% in ERr 731 vs. 76% in HRT) suggest that the severity of menopausal symptoms, which could influence the choice of treatment, was not a confounder. Finally, retrospective studies only allow conclusions to be drawn regarding associations and not causal effects.

In conclusion, the present study provides strong evidence that ERr 731 use is not associated with an increased risk of breast cancer diagnosis compared to both non-users and HRT users. The similarity in breast cancer incidence across different age groups further supports the safety of ERr 731 for women-seeking relief from menopausal symptoms. Given its favorable safety profile, ERr 731 may represent a viable alternative to HRT, particularly for women concerned about breast cancer risk.

## Data Availability

The data presented in this study are available on request from the corresponding author. Or the data are not publicly available due to privacy restrictions.
